# Prevalence of Anemia and Factors Associated With Handgrip Strength in Indonesian Elderly Population

**DOI:** 10.7759/cureus.25290

**Published:** 2022-05-24

**Authors:** Noorwati Sutandyo, Ikhwan Rinaldi, Nina K Sari, Kevin Winston

**Affiliations:** 1 Division of Hematology and Medical Oncology, Department of Internal Medicine, Dharmais National Cancer Hospital, Faculty of Medicine, Universitas Indonesia, Jakarta, IDN; 2 Division of Hematology and Medical Oncology, Department of Internal Medicine, Cipto Mangunkusumo National General Hospital, Faculty of Medicine, Universitas Indonesia, Jakarta, IDN; 3 Geriatric Medicine, Dharmais National Cancer Hospital, Faculty of Medicine, Universitas Indonesia, Jakarta, IDN; 4 Department of Internal Medicine, Cipto Mangunkusumo National General Hospital, Faculty of Medicine, Universitas Indonesia, Jakarta, IDN

**Keywords:** comorbidities, elderly, handgrip, hematology, anemia

## Abstract

Introduction: Anemia is a common blood disorder in the elderly which is associated with numerous poor medical outcomes. However, there is currently no study that assesses anemia prevalence of the Indonesian elderly population aged ≥60 years old in multiple provinces and analyzes its association with handgrip muscle strength using a large sample size.

Aim: We aimed to elucidate the prevalence of anemia and analyze factors associated with handgrip strength in elderly.

Method: This was a cross-sectional study using data from the Indonesian Family Life Survey-5 (IFLS-5). All participants aged ≥60 years old were included in this study. Exclusion criteria were: (1) respondents who refused to take health measurements (hemoglobin (Hb) level, handgrip strength, weight, stature, and waist circumference); (2) respondents with incomplete or missing data; (3) respondents with history of stroke; and (4) respondents with history of pain, swelling, inflammation, injury, and surgery on one or both hands within the last 6 months. The dependent variable for this study was handgrip strength. Subjects were classified as weak if the handgrip strength was <28 kg for men and <18 kg for women based on classification from the Asian Working Group for Sarcopenia (AWGS) 2019. The independent variables were Hb level, gender, age, body mass index (BMI), waist circumference, smoking history, comorbidities, and current use of drug therapies. Based on WHO standard, male and female participants with Hb less than 13 g/dL and 12 g/dL, respectively, are defined as anemic. Statistical analyses used included correlation, bivariate logistic regression, and multivariate logistic regression.

Result: A total of 3192 individuals were selected for analysis. Overall, 38.8% of participants had anemia, and the prevalence of anemia increases with age. A total of 56.30% of participants aged  ≥80 years had anemia. There was a positive correlation between Hb level and handgrip strength in the Indonesian elderly population (r: 0.349; p value: <0.001). Multivariate analysis showed that anemia was significantly associated with weak handgrip strength (OR: 1.557; 95% CI: 1.314-1.846; p value: <0.001).  Age ≥ 80 years (OR: 5.234), age 70-79 years (OR: 3.152), low BMI (OR: 1.827), and hypertension (OR: 1.340) were associated with weak handgrip strength in multivariate analysis.

Conclusion: The prevalence of anemia in the Indonesian elderly was 38.8% and anemia was associated with weak handgrip strength. The association of anemia with weak handgrip strength is more pronounced in males and the elderly aged ≥80 years.

## Introduction

Indonesia, the fourth most populous country, is experiencing an increase in life expectancy [1,2]. By the year 2050, it is predicted that the Indonesian aging population will be about 25% (around 74 million) of the total Indonesian population [3]. Thus, Indonesia must be ready to tackle common medical issues related to the elderly population such as anemia and frailty [4,5].

A systematic review conducted by Gaskell et al. showed that the weighted mean anemia prevalence was 17% (3-50%) in the elderly [4]. Anemia is universally recognized as an important risk factor for numerous adverse clinical outcomes in the elderly such as mortality, cardiovascular disease, dementia, and cognitive impairment [6-9]. Another clinical significance of anemia is its association with the reduction of handgrip strength and other functional outcomes [10-15]. It is speculated that anemia causes diminished muscular oxygenation and reduced muscle strength through impaired oxygen delivery [11,16]. The decline of exercise tolerance in anemia may then further reduce muscle mass through disuse atrophy [17].

Nevertheless, many of these studies were conducted in the Caucasian populations, which have a different body composition compared to the Asian populations [18,19]. As an example, members of the Asian population have a smaller body size, higher adiposity proportion, different socioeconomic backgrounds, and palm length [18-21]. These differences were shown to impact many medical outcomes [20,22]. Furthermore, there is a relative paucity of data regarding anemia prevalence in Asian countries, especially in Southeast Asia.

Hence, we aimed to elucidate the prevalence of anemia in the Indonesian elderly population and to analyze its association with handgrip strength in the elderly. To the best of our knowledge, there is currently no study that assesses the anemia prevalence of the Indonesian elderly population in multiple provinces and analyzes its association with handgrip muscle strength using a large sample size.

## Materials and methods

Setting

The Indonesian Family Life Survey (IFLS) is a longitudinal large-scale survey based on samples of households living in 13 provinces from three different islands in Indonesia which represented 83% of the Indonesian population [23,24]. There are four provinces from Sumatra Island (North Sumatra, West Sumatra, South Sumatra, and Lampung) and five provinces from Java Island (DKI Jakarta, West Java, Central Java, DI Yogyakarta, East Java). Furthermore, the provinces also included Bali, West Nusa Tenggara, South Kalimantan, and South Sulawesi. IFLS is currently the only multipurpose population-based survey with a large sample size in Indonesia that measured health outcomes.

In IFLS, a multistage stratified sampling design was used, which was conducted at the provincial level initially [23]. Subsequently, the sampling was conducted randomly within the provinces. Additional information on IFLS has been described in previous studies using data from IFLS [24-27]. This article was previously posted to the Research Square preprint server on December 1, 2021.

Statement of ethics

The procedures of IFLS surveys were reviewed and approved by institutional review boards (IRBs) in both United States and Indonesia [23]. In the United States, the IRBs responsible for ethical review of IFLS was Research and Development (RAND) corporation, a nonprofit think tank. Meanwhile, in Indonesia, the IFLS-5 study was approved by IRBs at Universitas Gadjah Mada (UGM). The protocol approval number given by RAND’s Human Subjects Protection Committee (RAND’s IRB) for IFLS-5 was s0064-06-01-CR01.

Data availability

The datasets supporting the conclusions of this article are available from the RAND website: https://www.rand.org/well-being/social-and-behavioral-policy/data/FLS/IFLS.html.

Study design

This study was a multicenter, non-interventional, cross-sectional study using data from IFLS5 that was conducted in late 2014 and early 2015 in 13 provinces of Indonesia. The household response rate for IFLS5 was very high (92%) [23,24]. The dependent variable for this study was handgrip strength. The independent variables were hemoglobin level, gender, age, body mass index (BMI), waist circumference, smoking history, comorbidities, and current use of drug therapies.

The inclusion criteria used in this study were respondents aged ≥60 years old as in Indonesia, the elderly is defined as age 60 years and above [28]. Exclusion criteria were (1) respondents who refused to take health measurements (hemoglobin (Hb) level, handgrip strength, weight, stature, and waist circumference); (2) respondents with incomplete or missing data; (3) respondents with a history of stroke; and (4) respondents with a history of pain, swelling, inflammation, injury, and surgery on one or both hands within the last 6 months.

Hemoglobin level

A Hb test was performed using capillary blood drawn from a finger prick. The measurement was performed using a HemoCue handheld meter (model Hb201+; HemoCue Holding AB, Ängelholm, Sweden) together with its respective HB201 microcuvettes. The finger sticks lancets manufactured by Hospital and Home Care were used. Meanwhile, the dried blood spot cards used were Whatman® 903 Protein Saver Cards containing five half-inch circles with each circle capable of holding 75 to 80 μL of the sample [29].

Based on WHO standard, male and female participants with Hb less than 13 g/dL and 12 g/dL, respectively, are defined as anemic [30].

Handgrip strength

Handgrip strength on each hand was measured twice using a Baseline Smedley Spring type dynamometer. The dynamometer was calibrated daily. Trained personnel instructed the study participants to hold and squeeze the handle of the dynamometer as firmly as they could. The measurement begins with the dominant hand and continues with an alternating hand, with a resting period in between measurements. The value for handgrip strength used for this study was the average of both left and right hands, each measured twice. Subjects were classified as weak if the handgrip strength was <28 kg for men and <18 kg for women based on classification from the Asian Working Group for Sarcopenia (AWGS) 2019 [18].

Confounders

Confounders included in this study were age, sex, BMI, waist circumference, history of smoking, comorbidities, history of taking anemia medicine, history of taking hypertension medicine, history of taking diabetes medicine, and history of taking cholesterol medicine. The stature of subjects was recorded to the nearest millimeter using a Seca plastic height board (model 213). Meanwhile, the weights of subjects were measured using the Camry model EB1003 scale to the nearest tenth of a kilogram. Subsequently, BMI was calculated as weight in kg divided by stature in meter square. The BMI was classified based on Western Pacific Region of World Health Organization criteria which are: (1) <18.5 kg/m^2^ as underweight; (2) 18.5 to 22.9 kg/m^2^ as normal weight; (3) 23.0 to 24.9 kg/m^2^ as overweight; and (4) ≥25 kg/m^2^ as obese [31].

Waist circumference was based on cutoffs of ≥80 cm for women and ≥90 cm for men. The comorbidities were heart disease, diabetes, hypercholesterolemia, hypertension, kidney disease, and tuberculosis. Comorbidities were assessed using the question “Have a doctor/paramedic/nurse/midwife ever told you had…. (Comorbidities mentioned above)”. This study also investigated whether the participant had been taking drug therapies for anemia, hypertension, diabetes, and hypercholesterolemia. Participants were categorized into yes and no groups based on whether they take the medicine or not.

Statistical analysis

All statistical analyses were performed using SPSS statistical software version 21.0 (IBM Corp, Armonk, NY, USA) with statistical significance defined as p < 0.05.

In this study, categorical variables reported as percentages were used for characteristics of our study population. Prevalence of anemia and weak handgrip strength were calculated as percentages. Sex-specific and age-specific anemia prevalence were also calculated. Differences in prevalence of anemia between participants aged ≥80 years old with those aged <80 years old were assessed using Chi-square (χ^2^) test. Correlation analysis using Pearson’s correlation would be used if the data (plural) were normally distributed. If not, a Spearman correlation test would be used. The normality of data was assessed using the Kolmogorov-Smirnov test or Shapiro-Wilk test.

Logistic regression was conducted for bivariate and multivariate analyses of risk factors associated with weak handgrip strength. Subsequently, the Hosmer-Lemeshow test was used to determine the goodness of fit for the multivariate model. A multicollinearity test was also conducted with multicollinearity defined as variable inflation factor (VIF) >5. The multivariate analysis in this study employed a backward elimination model-building process.

Subgroup analysis was then conducted to explore the association between anemia and weak handgrip strength based on gender and age (60-69 years, 70-79 years, and ≥ 80 years)

## Results

**Figure 1:**
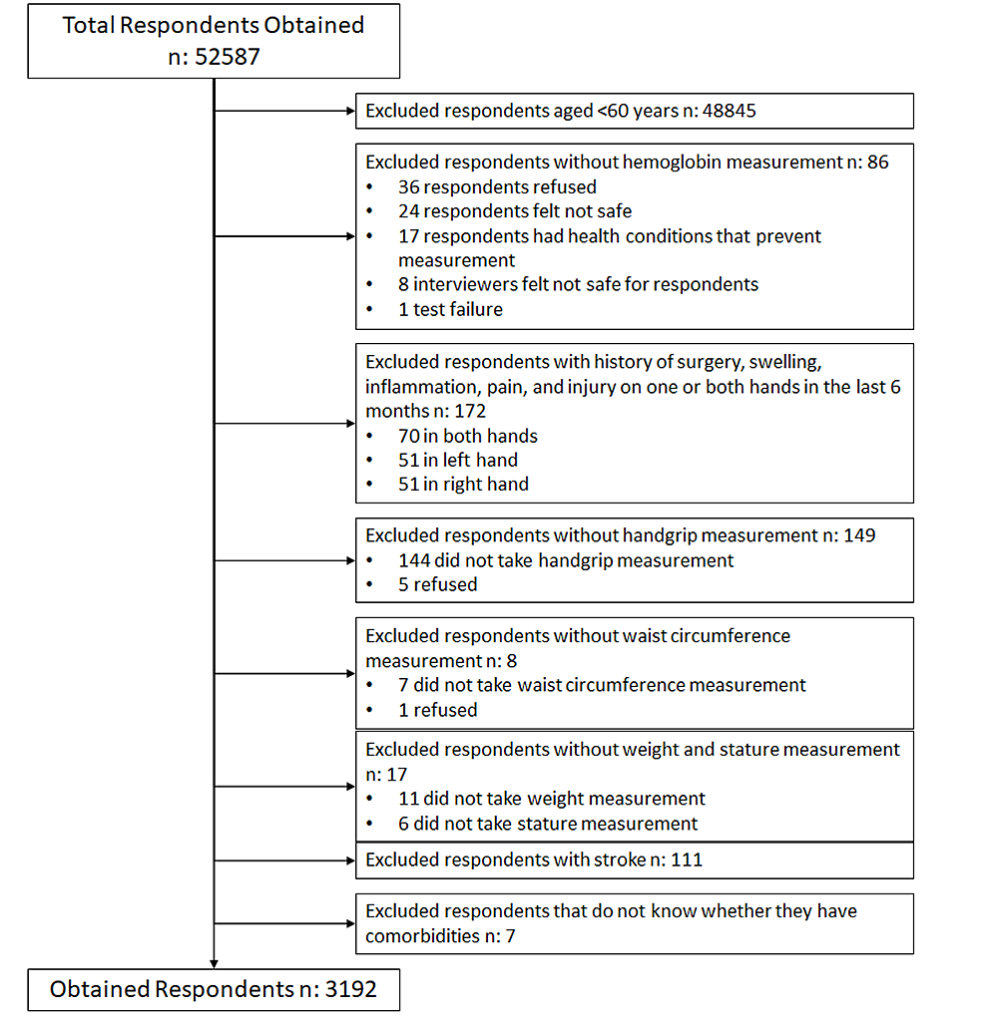
Flowchart of participants selection

In total, 52,587 unique subjects were obtained after combining multiple datasets of IFLS5. Of these, 3742 participants aged ≥60 years old were selected. Subsequently, 86 individuals without Hb measurement and 172 individuals with a history of pain, swelling, inflammation, injury, and surgery on one or both hands within the last 6 months were excluded. Another 174 individuals were excluded due to missing data in handgrip, waist circumference, and weight or stature measurements. Additionally, 111 individuals with stroke were excluded. Finally, seven individuals that did not know whether they have comorbidities were excluded. Hence, a total of 3192 individuals were selected for analysis (Figure 1).

Characteristics of participants are summarized in Table 1. Overall, there were 1959 participants aged 60-69 years (61.4%), 954 participants aged 70-79 years (29.9%), and 279 participants aged ≥80 years (8.7%). The majority of participants had normal BMI (42.1%) and normal waist circumference (58%). The highest comorbidities observed was hypertension with 28.5% of participants having hypertension. About 44.9% of participants had a smoking history.

**Table 1:** Characteristics of the study participants

Variables	Category	All Gender (n: 3192)	Males (n: 1518)	Females (n: 1674)
		n (%)	n (%)	n (%)
Anemia	Yes	1238 (38.8)	593 (39.1)	645 (38.5)
	No	1954 (61.2)	925 (60.9)	1029 (61.5)
Handgrip strength	Weak strength	2192 (68.7)	1035 (68.2)	1157 (69.1)
	Normal strength	1000 (31.3)	483 (31.8)	517 (30.9)
Age	≥ 80 years	279 (8.7)	141 (9.3)	138 (8.2)
	70-79 years	954 (29.9)	443 (29.2)	511 (30.5)
	60-69 years	1959 (61.4)	934 (61.5)	1025 (61.2)
BMI	Obese	732 (22.9)	232 (15.3)	500 (29.9)
	Overweight	478 (15)	212 (14)	266 (15.9)
	Underweight	638 (20)	333 (21.9)	305 (18.2)
	Normal	1344 (42.1)	741 (48.8)	603 (36)
Waist circumference	High	1340 (42)	294 (19.4)	1046 (62.5)
	Normal	1852 (58)	1224 (80.6)	628 (37.5)
Smoking history	Yes	1432 (44.9)	1213 (79.9)	219 (13.1)
	No	1760 (55.1)	305 (20.1)	1455 (86.9)
Heart disease	Yes	133 (4.2)	61 (4)	72 (4.3)
	No	3059 (95.8)	1457 (96)	1602 (95.7)
Hypertension	Yes	911 (28.5)	350 (23.1)	561 (33.5)
	No	2281 (71.5)	1168 (76.9)	1113 (66.5)
Hypercholesterolemia	Yes	196 (6.1)	76 (5)	120 (7.2)
	No	2996 (93.9)	1442 (95)	1554 (92.8)
Diabetes	Yes	173 (5.4)	88 (5.8)	85 (5.1)
	No	3019 (94.6)	1430 (94.2)	1589 (94.9)
Kidney disease	Yes	48 (1.5)	29 (1.9)	19 (1.1)
	No	3144 (98.5)	1489 (98.1)	1655 (98.9)
Tuberculosis	Yes	46 (1.4)	31 (2)	15 (0.9)
	No	3146 (98.6)	1487 (98)	1659 (99.1)
Taking anemia medicine	Yes	50 (1.6)	22 (1.4)	28 (1.7)
	No	3142 (98.4)	1496 (98.6)	1646 (98.3)
Taking hypertension medicine	Yes	270 (8.5)	106 (7)	164 (9.8)
	No	2922 (91.5)	1412 (93)	1510 (90.2)
Taking diabetes medicine	Yes	91 (2.9)	46 (3)	45 (2.7)
	No	3101 (97.1)	1472 (97)	1629 (97.3)
Taking cholesterol medicine	Yes	61 (1.9)	19 (1.3)	42 (2.5)
	No	3131 (98.1)	1499 (98.7)	1632 (97.5)

The prevalence of anemia can be seen in Table 2. Overall, 38.8% of participants aged ≥60 years had anemia. When adjusted by age, the prevalence of anemia is lowest in age 60-64 (28.8%), but it slowly increases with age, resulting in participants aged ≥80 years old having the highest prevalence of anemia (56.3%). Chi-square analysis showed that there was a statistically significant difference in anemia prevalence between subjects aged ≥80 years and subjects aged <80 years (Table 6 in Appendices). Using handgrip criteria from AWGS, a total of 68.7% of participants had weak handgrip [18]. The prevalence of weak handgrip also increased with age.

**Table 2:** Prevalence of anemia and weak handgrip strength based on age and gender

Variables	Gender	Age					
		All age	60-64	65-69	70-74	75-79	≥80
		(n: 3192)	(n: 1216)	(n: 743)	(n: 636)	(n: 318)	(n: 279)
Anemia	All gender	38.80%	31.50%	37.70%	41.50%	48.40%	56.30%
	Male only	39.10%	28.80%	39.40%	42.30%	52.00%	59.60%
	Female only	38.50%	33.9%	36.10%	40.80%	45.20%	52.90%
Weak handgrip strength	All gender	68.70%	52.80%	67.20%	81.60%	87.70%	90.70%
	Male only	68.20%	51.5%	65.6%	81.9%	90%	91.5%
	Female only	69.10%	54%	68.6%	81.3%	85.7%	89.9%

The variables of Hb, age, BMI, and waist circumference were analyzed for normality test using Kolmogorov-Smirnov test and Shapiro-Wilk test, which showed non-normal data distribution for all these variables. Thus, Spearman correlation was used for the correlation test. Based on correlations tests, Hb, BMI, and waist circumference were positively correlated with handgrip strength (Table 3). In subjects with age ≥80 years, the correlation between Hb and handgrip strength was still positive (r: 0.224; p value: <0.001) Meanwhile, age was inversely correlated with handgrip strength and the correlation was stronger in males.

**Table 3:** Correlation test results

Variables	All patient (n: 3192)		Male only (n: 1518)		Female only (n: 1674)		Age 60-79 only (n: 2913)		Age ≥80 only (n: 279)	
	Correlation coefficient	P value	Correlation coefficient	P value	Correlation coefficient	P value	Correlation coefficient	P value	Correlation coefficient	P value
Hemoglobin level-handgrip strength	0.349	<0.001	0.246	<0.001	0.131	<0.001	0.349	<0.001	0.224	<0.001
Age-handgrip strength	-0.319	<0.001	-0.425	<0.001	-0.383	<0.001	-0.271	<0.001	-0.104	0.084
BMI-handgrip strength	0.094	<0.001	0.289	<0.001	0.251	<0.001	0.061	0.001	0.063	0.296
Waist circumference-handgrip strength	0.048	<0.001	0.238	<0.001	0.178	<0.001	0.026	0.168	0.045	0.452

Bivariate and multivariate analyses are shown in Table 4. Hosmer-Lemeshow test to assess the quality of the multivariate analysis models showed a p value of 0.424 (χ^2^: 8.098 and degree of freedom: 8). Therefore, there is no statistically significant difference between observed handgrip strength status and expected handgrip status from the multivariate model, indicating a well-calibrated model. The multicollinearity test of the variables used for multivariate analysis showed no presence of multicollinearity. The multicollinearity tests can be seen in Table 7 (in Appendices). The multivariate analysis showed that anemia (OR: 1.557), older age (OR: 5.234 for ≥ 80 years and OR: 3.152 for 70-79 years), low BMI (OR: 1.827), and hypertension (OR: 1.340) were associated with weak handgrip. Meanwhile, history of taking anemia medicine was protective (OR: 0.539; 95% CI: 0.294-0.989; p value: 0.046).

**Table 4:** Bivariate and multivariate analysis between variables and weak handgrip strength (n: 3192)

Variable	Category	Bivariate Analysis			Multivariate Analysis		
		Odds ratio	95% CI	p value	Adjusted Odds ratio	95% CI	p value
Anemia	Yes	1.904	1.621-2.236	<0.001	1.557	1.314-1.846	<0.001
	No	Reference	-	-	Reference	-	-
Age	≥ 80 years	6.976	4.613-10.549	<0.001	5.234	3.438-7.969	<0.001
	70-79 years	3.667	3.022-4.451	<0.001	3.152	2.584-3.843	<0.001
	60-69 years	Reference	-	-	Reference	-	-
Sex	Male	0.958	0.824-1.112	0.570	-	-	-
	Female	Reference	-	-	Reference	-	-
BMI	Obese	0.547	0.454-0.660	<0.001	0.696	0.570-0.850	<0.001
	Overweight	0.750	0.602-0.934	<0.001	0.861	0.684-1.085	0.204
	Low	2.134	1.681-2.710	<0.001	1.827	1.424-2.345	<0.001
	Normal	Reference	-	-	Reference	-	-
Waist circumference	High	0.579	0.497-0.673	<0.001	-	-	-
	Normal	Reference	-	-	Reference	-	-
Smoking history	Yes	1.052	0.905-1.223	0.508	-	-	-
	No	Reference	-	-	Reference	-	-
Hypertension	Yes	1.185	1.001-1.402	0.048	1.340	1.116-1.609	0.002
	No	Reference	-	-	Reference	-	-
Hypercholesterolemia	Yes	0.562	0.419-0.752	<0.001	0.764	0.557-1.046	0.093
	No	Reference	-	-	Reference	-	-
Heart disease	Yes	1.063	0.728-1.552	0.750	-	-	-
	No	Reference	-	-	Reference	-	-
Diabetes	Yes	0.786	0.571-1.081	0.139	-	-	-
	No	Reference	-	-	Reference	-	-
Kidney disease	Yes	0.692	0.386-1.241	0.217	-	-	-
	No	Reference	-	-	Reference	-	-
Tuberculosis	Yes	1.653	0.817-3.344	0.162	-	-	-
	No	Reference	-	-	Reference	-	-
Take medicine for anemia	Yes	0.488	0.279-0.855	0.012	0.539	0.294-0.989	0.046
	No	Reference	-	-	Reference	-	-
Take medicine for hypertension	Yes	0.888	0.682-1.157	0.379	-	-	-
	No	Reference	-	-	Reference	-	-
Take medicine for diabetes	Yes	0.759	0.493-1.168	0.209	-	-	-
	No	Reference	-	-	Reference	-	-
Take medicine for hypercholesterolemia	Yes	0.749	0.444-1.265	0.280	-	-	-
	No	Reference	-	-	Reference	-	-

We also conducted a subgroup analysis based on age (Table 5). Based on the analysis, the ORs for weak handgrip strength were highest in participants aged ≥ 80 years (OR: 2.662; 95% CI: 1.143-6.203; p value: 0.023). Additionally, participants of the male gender had higher odds ratios for weak handgrip strength when compared with the female gender.

**Table 5:** Subgroup analysis for association between anemia and weak handgrip strength based on age and gender *Adjusted for sex, BMI, smoking history, waist circumference, diabetes, heart disease, hypercholesterolemia, hypertension, kidney disease, tuberculosis, take medicine for anemia, take medicine for hypertension, take medicine for diabetes, take medicine for hypercholesterolemia. ^Adjusted for age, BMI, smoking history, waist circumference, diabetes, heart disease, hypercholesterolemia, hypertension, kidney disease, tuberculosis, take medicine for anemia, take medicine for hypertension, take medicine for diabetes, take medicine for hypercholesterolemia.

Variable	Category	Odds Ratio for Weak Handgrip Strength	Lower 95% CI	Upper 95% CI	P value
Age^*^	60-69	1.569	1.286	1.914	<0.001
	70-79	1.395	0.975	1.995	0.068
	≥ 80	2.662	1.143	6.203	0.023
Gender^	Male	1.826	1.418	2.352	<0.001
	Female	1.336	1.059	1.684	0.014

## Discussion

In this study, we found that 38.8% of participants aged ≥60 years (n: 3192) had anemia and a total of 68.7% participants had weak handgrip. We also observed that the prevalence of anemia and weak handgrip increase with age. There was a positive correlation between Hb level and handgrip strength in the Indonesian elderly population (r: 0.349; p value: 0.000). Multivariate analysis showed that anemia was significantly associated with weak handgrip strength (OR: 1.557; 95% CI: 1.314-1.846; p value: <0.001). Furthermore, the OR for age ≥ 80 years is higher (OR: 5.234; 95% CI: 3.438-7.969; p value: <0.001) than OR for age 70-79 years (OR: 3.152; 95% CI: 2.584-3.843; p value: <0.001). This study confirms that the Indonesian elderly have high prevalence of anemia.

Anemia is often perceived as a benign condition due to the aging process. Recently, this paradigm is challenged by studies showing that even mild anemia is a risk factor for adverse clinical outcomes in the elderly [6-9]. This current study is the first Indonesian study with a large sample that assesses the prevalence of anemia in the elderly. We observed that 38.8% of participants aged ≥60 years had anemia based on the definition of anemia by WHO (Hb less than 12 g/dL in women and less than 13 g/dL in men). This is similar to the study by Hidayat et al. using 118 elderly participants living in nursing homes [32]. According to a systematic review by Gaskell et al. of 45 studies, the mean prevalence of anemia in older adults was 17% (3-50%) [4]. Therefore, our results confirm that anemia is also common in the Indonesian elderly population and has a higher prevalence, albeit within the range of the systematic review by Gaskell et al. [4]. Furthermore, the anemia prevalence is similar to the prevalence of Malaysia, a neighboring country that has similar ethnicities, with anemia prevalence of 35.3% [33]. Other studies in north-eastern Thailand, Philippines, and India showed anemia prevalence of 48%, 21.6%, and 68.7%, respectively in the elderly [34-36]. However, Indonesia and all of these countries are in contrast with Singapore and Taiwan which had a lower anemia prevalence of 15.2% and 18.8%, respectively [37,38]. The difference in prevalence between countries may be caused by heterogeneity in living conditions, elderly healthcare, health problems, and socioeconomic factors.

The age-associated increase of anemia in our study appears to be more pronounced in males than females. By the age of ≥65 years old, males have a higher prevalence of anemia than females in our study. This observation is similar to data from Third National Health and Nutrition Examination Survey (NHANES III) in the USA [39]. Other studies also observe this phenomenon [4,40-43]. The difference is that in the data by NHANES III, anemia became more common in males starting from age 75 while the anemia in our study started to become more common from age 65 years old.

We hypothesize that the prevalence of anemia in the Indonesian elderly may be indirectly caused by economic status and poverty since a significant number of Indonesians are living below the poverty line. However, studies are needed to analyze the role of economic status and poverty. Poverty may impact the ability to access necessary animal-based food which contains iron to maintain Hb. Data from NHANES III showed that around one-third of the anemic elderly had deficiencies in iron, folate, and cobalamin [39,44]. Additionally, a study specific to the Indonesian elderly in East Java found that anemia in the elderly is related to lower consumption of folic acid and higher consumption of coffee and tea due to the role of tannins as iron absorption inhibitors [45]. Other possible causes of anemia in the elderly include chronic kidney disease (CKD), chronic inflammation, and myelodysplastic syndrome [44]. Unfortunately, the datasets we obtained for this study did not have kidney function, marker of inflammations, and bone marrow examinations which made analyses of these causes impossible. Furthermore, there are currently no published studies in Indonesia on the elderly Indonesian population that analyze the role of CKD, chronic inflammation, and myelodysplastic syndrome in anemia.

Prevalence of weak handgrip in Indonesian elderly

Using criteria from AWGS 2019 that was based from eight Asian cohorts aged ≥65 years, we observed that a total of 68.7% participants in our study had weak handgrip [18,46]. A study by Ashdown-Franks et al. using samples of individuals aged ≥ 50 years from six low- and middle-income countries showed weak handgrip prevalence of 47.4% [47]. Meanwhile, a study by Gi et al. in Korea showed a weak handgrip prevalence of 12.5% [48]. However, it should be noted that these studies differ in weak handgrip strength definitions, economic income, and racial population.

A recent study conducted in our institution using 164 elderly patients showed a weak handgrip prevalence of 67.1% [49]. However, despite a similar prevalence of weak handgrip with this study, it may not be comparable since the study used Jamar hydraulic dynamometer [49]. Here, we used a Smedley spring-type dynamometer for handgrip measurement which is the commonest used device in Asia for measuring handgrip strength [50]. The second commonest used device is Jamar hydraulic dynamometer [50]. According to AWGS 2019, the handgrip measurement from Jamar hydraulic dynamometer may be higher when compared with the Smedley spring-type dynamometer [18]. Thus, using different dynamometer may impact the prevalence of weak handgrip strength in a study, however, there is currently no available specific cutoffs made for these dynamometers [18].

Anemia causes a reduction in the oxygen-carrying capacity of the blood to organs, including to muscle tissue [11,16]. This impairment of oxygen delivery can result in decreased aerobic capacity, decreased physical strength, and sarcopenia, and further reduce the already declined physiologic reserve in the elderly [11,12,16,51]. It is also possible that elderly subjects with anemia are less active resulting in muscle disuse [52]. Additionally, both muscle strength and muscle mass appeared to be lower in anemic elderly [11,13,53]. However, it is believed that there are still unknown pathophysiological processes of anemia in the elderly. Further studies to elucidate the complete mechanisms of how anemia affects the elderly should be conducted.

Factors associated with weak handgrip strength

The correlation tests in this study showed a positive correlation between Hb level and handgrip strength in the Indonesian elderly population which is consistent with other studies [11,14,15,48,54,55]. Studies using elderly population of ≥80 years old also demonstrated association between anemia with handgrip strength [56,57]. Similarly, we also observed that the association of anemia with weak handgrip was more pronounced in the oldest old group and in the male group. Other studies have also shown the role of anemia in other medical outcomes such as cognitive declines and mortality [9,17,58,59]. Thus, anemia is clinically important in elderly due to poor medical outcomes associated with it. Further supporting this is the data showing that even low Hb level in normal range cause higher risk of frailty [60].

Based on this, it may be considered that the oldest old group should be screened for anemia and given prompt treatment. We observe that participants taking medicine had low ORs for weak handgrip, however, the 95% CI was very wide and further information on the type of anemia medicine and the dose were unavailable. A study by Amano et al. showed that ageism can affect medical care in the elderly [61]. Thus, some elderly subjects may not be given anemia medicine despite having anemia and this may influence the 95% CI of taking anemia medicine variable in this study. Indeed, further studies to analyze how ageism affects anemia treatment and medical outcomes should be conducted.

Another important finding in this study is that the association of anemia with weak handgrip was more pronounced in the oldest old group and in the male group. Nevertheless, despite these significant findings, it is very difficult to directly declare that anemia is a causal risk factor for weak handgrip due to observational nature of this study and the possibility of anemia being a marker for other medical conditions. For example, anemia is closely related to chronic inflammation which is observed in sarcopenia, hence, may indicate a bidirectional relationship between anemia and sarcopenia [62-64].

Our study also found that low BMI is associated with weak handgrip strength. This is in line with a study by Lad et al. [65]. However, in contrast with their study, we do not find an association between high BMI and handgrip strength. This difference may be explained due to differences in study populations.

Study limitations

Despite bridging the scientific gap between anemia and handgrip strength in the Indonesian elderly, the current study has several limitations. First, this study is a cross-sectional study that is unable to elucidate a causal relationship between the variables and weak handgrip. Second, this study did not analyze the impact of dietary factors among the subjects on anemia. The type of anemia also could not be identified because of the insufficient laboratory and lack of nutritional status data.

## Conclusions

The prevalence of anemia in the Indonesian elderly population was 38.8%. When adjusted by age, the prevalence of anemia is lowest in age 60-64 (28.8%), but it slowly increases with age, resulting in participants aged ≥80 years old having the highest prevalence of anemia (56.3%). Furthermore, anemia was significantly associated with weak handgrip strength in the Indonesian elderly population. This association was stronger for males and elderly aged ≥80 years old. Potential mechanisms of how anemia affects handgrip strength include reduction in the oxygen-carrying capacity of the blood muscle tissue leading to impaired oxygenation and subsequent disuse atrophy.

## Appendices

**Table 6:** Chi-square analysis for anemia proportion

	Anemia Status		Total	P value (Continuity Correction)
	Anemia	No Anemia		
Age ≥80 years old	157 (56.3%)	122 (43.7%)	279 (100%)	<0.001
Age <80 years old	1081 (37.1%)	1832 (62.9%)	2913 (100%)	
Total	1238 (61.2%)	1954 (38.8%)	3192 (100%)	

**Table 7:** Multicollinearity tests VIF: variable inflation factor

Variable	Collinearity Statistics	
	Tolerance	VIF
Anemia	0.967	1.034
Age	0.928	1.077
Sex	0.495	2.021
BMI	0.654	1.528
Waist circumference	0.548	1.826
Smoking history	0.534	1.874
Hypertension	0.812	1.231
Hypercholesterolemia	0.847	1.180
Heart disease	0.947	1.056
Diabetes	0.597	1.675
Kidney disease	0.977	1.023
Tuberculosis	0.976	1.024
Take medicine for anemia	0.971	1.030
Take medicine for hypertension	0.811	1.233
Take medicine for diabetes	0.601	1.665
Take medicine for hypercholesterolemia	0.865	1.156
